# Uncovering biosecurity gaps: risk factors for PRRSV seropositivity in Costa Rican pig farms identified through machine learning

**DOI:** 10.1186/s40813-026-00495-4

**Published:** 2026-02-21

**Authors:** Ronald Meléndez-Arce, Emily Jiménez-Loaiza, Berta Leiva-Bonilla, Juan Carlos Venegas-Soto, Milania Rocha-Palma, Arie Van Nes, Arjan Stegeman, Hans Vernooij, Juan José Romero-Zúñiga

**Affiliations:** 1 Department Population Health Sciences, Utrecht University, Utrecht, The Netherlands; 2 Programa de Investigación en Medicina Poblacional, Universidad Nacional, Heredia, Costa Rica; 3 Cooperativa de Productores de Leche R.L., San José, Costa Rica; 4 Ministerio de Salud, San José, Costa Rica; 5 Facultad de Farmacia, Universidad de Costa Rica, San José, Costa Rica

**Keywords:** PRRSV, Biosecurity, Pig farms, Costa Rica, LASSO, Random Forest, Seroprevalence, Controlled exposure, Risk factors, Machine learning

## Abstract

**Background:**

Porcine Reproductive and Respiratory Syndrome Virus (PRRSV) continues to impose significant economic losses on pig production globally. In Costa Rica, where the virus is endemic, there is limited knowledge of the farm-level risk factors influencing PRRSV spread. This study aimed to identify biosecurity factors associated with PRRSV seroprevalence in Costa Rican pig farms.

**Methods:**

A cross-sectional survey was conducted on 21 pig farms across Costa Rica. Data on farm management and biosecurity practices were collected using a structured questionnaire and linked to PRRSV seroprevalence data from a companion study. Logistic regression, and machine learning methods like LASSO (Least Absolute Shrinkage and Selection Operator), and Random Forest models were used to identify significant risk factors associated with herd-level PRRSV positivity.

**Results:**

Three key risk factors were consistently identified by both LASSO and Random Forest models: historical controlled exposure to PRRSV, restrictions on employee access to the farm, and restrictions on employee visits to other pig farms. Additional risk factors identified included topography, disinfection practices for transport vehicles, sanitation measures for visitors, boot and clothing protocols, and feedback procedures. Farms with a history of controlled exposure had an odds ratio of 90 (95% CI: 7.6–3,550) for being PRRSV-positive.

**Conclusion:**

The findings underscore the importance of internal and external biosecurity measures, particularly in relation to personnel movement and intentional exposure practices. Modeling approaches such as LASSO and Random Forest provided complementary insights into PRRSV risk factors in a tropical production setting. These insights can guide tailored interventions to reduce PRRSV transmission in Costa Rica and similar regions.

**Supplementary Information:**

The online version contains supplementary material available at 10.1186/s40813-026-00495-4.

## Background

Porcine reproductive and respiratory syndrome (PRRS) was first reported in the United States (USA) in 1987 [1] and in Europe in 1990 [2]. Since then, the disease has spread widely across in many pig-producing countries, affecting both reproduction and respiratory health in pigs [3, 4]. Porcine Reproductive and Respiratory Syndrome Virus (PRRSV), the causative agent, is considered one of the most economically significant pathogens in pig farming globally. In the United States alone, annual losses to PRRS were estimated at $664 million in 2013, rising $1.2 billion by 2024 [5]. In Costa Rica, there is no estimate of the real costs, but a model predicted the costs to be US$240 - US $890 per sow for a farrow-to-finish farm with PRRS-low, medium and high scenarios [6].

Given the high economic impact, preventing PRRSV-infection is of paramount importance. This can be achieved through a combination of vaccination and enhanced biosecurity measures [7]. While vaccination helps to reduce viraemia and clinical signs [8], biosecurity measures include several actions applied in animal health to prevent introduction and spread (external) and within transmission (internal) of infectious diseases. Enhancing both biosecurity measures may be done by limiting both direct and indirect contacts between pigs.

In Costa Rica, PRRSV was first reported in 1996 [9] and has since had a major impact on the national pig industry, causing reproductive failures and respiratory illness [10]. A recent study reported a PRRSV seroprevalence of 44% in Costa Rican farms [11], consistent with findings from other regions in Latin America [12]. Controlling PRRSV remains one of the key challenges in Costa Rican pig production. Understanding and managing risk factors associated with virus introduction (external biosecurity) [13], and within-farm transmission (internal biosecurity) are crucial for effective control and prevention [14, 15]. Several studies have identified important risk factors for PRRSV infection [13–15], including importation of live pigs or semen, movement of people, contaminated clothing and boots, shared needles, insect vectors (mosquitoes/flies), transport vehicles and aerosol transmission. However, it remains unclear to what extent these risk factors are relevant for Costa Rican farms compared to those in the US or Europe.

This study aimed to investigate the association between PRRSV-infection herd status and both external and internal biosecurity risk factors on pig farms in Costa Rica. The findings are expected to benefit not only Costa Rican producers, but also pig producers in other tropical and subtropical countries seeking to control PRRSV more effectively.

## Methods

### Study design and population

The total number of farms in Costa Rica affiliated to the National Chamber of Pig Farmers is 87. A cross-sectional study was conducted on 21 farrow-to-finish pig farms across the country, selected based on stratified sampling regarding herd size using the nationwide SIREA system (Integrated System of Registration of Agricultural Establishments) of the National Service of Animal Health and on willingness. All participating farms included the three production phases: breeding, weaning, and growing/finishing and maintained reproductive and production records. Farms were stratified by herd size: Stratum 1 (large farms > 500 sows) and Stratum 2 (small farms, ≤ 500 sows). The geographical distribution of sampled farms across the country’s provinces is presented in Table 1.

**Table 1:** Distribution of sampled farms by stratum and province in Costa Rica

Province	Stratum 1	Stratum 2
San José	0	3
Alajuela	2	5
Heredia	0	1
Cartago	1	1
Limón	4	1
Guanacaste	0	1
Puntarenas	0	2

### Within-herd prevalence

The within-herd seroprevalence data used for this study was derived from a previously published survey [11]. In summary, 50–60 blood samples per farm were taken from 8–12-week-old piglets and of own replacement gilts, accounting for detecting 5% within herd prevalence with 95% confidence level. A farm was classified as PRRSV-positive if at least one sampled animal tested positive by [ELISA/PCR], regardless of age group or location within the herd. There was one farm with one positive sample, which was also positive in the retest. 52% of the farms (11/21) tested seropositive for PRRSV, with positive cases found in six of Costa Rica’s seven provinces.

### Questionnaire

A structured questionnaire based on the PADRAP tool (Production Animal Disease Risk Assessment Program) [16] was adjusted to the Costa Rican pig production context. The modified version included 155 questions: 31 addressing internal risk factors (IR) and 124 addressing external risk factors (ER) [17]. Variables were grouped into risk categories according to their relevance to PRRSV introduction and spread (Supplementary Material, Table 1). All data collectors (4 veterinarians, sometimes supported by an assistant) received standardized training in interviewing techniques, auditing and data quality assurance. After completion, questionnaires were reviewed for completeness and clarity. In case of inconsistencies or missing information, data were verified and corrected via phone calls, e-mails, or follow-up farm visits.

### Statistical analysis

The dependent variable was farm PRRSV serostatus, coded as binary: 1 = seropositive or 0 = seronegative. Numerical responses were categorized when exact values were unavailable (e.g., responses such as “greater than …”) or dichotomized using the median (low versus high).

Categories of categorical variables were merged when some levels contained few or no positive or negative cases, which could compromise statistical power and model stability, as shown in Supplementary Material, Table 2.

The association between biosecurity variables and PRRSV status was assessed in two steps. Firstly, univariable binary logistic regression was used to estimate odds ratios (OR) with 95% confidence interval [18]. Firth’s correction was applied when zero-cell counts were encountered. Secondly, based on the lowest AIC from the univariable models, 36 plausible variables were selected for multivariable models. These included a Random Forest model for classification [19, 20] and a Least Absolute Shrinkage and Selection Operator (LASSO) model using the glmnet package [21–23]. Both models are machine learning models and use regression by decreasing the variance to find an association between the dependent and independent variables. The factors that emerge from the models are not by default significant but may show some biological or clinical importance to the outcome. For the LASSO model [24], 10-fold cross-validation identified an optimal lambda value of 0.026, corresponding to the model with the lowest deviance. All analyses were performed in statistical package R version 4.2.3 [25]. Extra information on Random Forest and LASSO model is given in supplementary file “Statistics”.

## Results

Of the 226 variables analyzed, 36 were identified as promising based on their contribution to model performance. These variables either reduced the overall AIC or, if removed resulted in a higher AIC, indicating their significance in explaining variation in the dependent variable (Supplementary Material, Table 2).

Combined modeling using Random Forest and LASSO selected three risk factors associated with PRRSV positivity status (Table 2), two associated with internal and one associated with external biosecurity. Farms with a history of controlled (intentional) exposure of PRRSV had a higher risk of testing positive (OR = 91% CI: 7.6–3547). Conversely, farms requiring a change of clothing and boots between sites tended to have a lower risk (OR = 0.11, 95% CI: 0.00-1.04). Unexpectedly, farms without restrictions on employee had a lower risk (OR = 0.04, 95% CI: 0.00-0.41) compared to those imposing restrictions after specific hours.

Random Forest identified three additional variables as important: sanitation measures for employees and visitors entering the site, the farm’s topography (physical landscape), and the type of disinfectant used on vehicles transporting breeding animals. LASSO, on the other hand, identified several other potentially relevant factors, including clothing and boot protocols, the sow-to-employee ratio, the practice of feedback (feeding tissues from deceased pigs to stimulate immunity) prior introduction, and restrictions on employees visiting other swine farms.

Some variables, such as prior PRRSV status and sow-to-employee ratio, remained in the LASSO model despite confidence intervals including 1, as these still contributed to the overall model performance.

**Table 2:** Estimated odds ratios and 95% confidence intervals for positive PRRSV herd status, based on risk factors selected by Random Forest and LASSO models

Variable	Herd status for PRRSv		OR	95% CI			
	positive (*n* = 11)	negative (*n* = 10)		LL	UL		
**Selection by LASSO and Random Forest**							
Historical controlled exposure							
* No*	1	9	Ref.	-	-		
* Yes*	10	1	90.02	7.59	3547.50		
Employee restrictions on visits to other swine production facilities							
* No restrictions*	6	2	Ref.	-	-		
* Not Applicable (farm has no employees)*	1	0	1.15	0.03	35.86		
* Visits to other swine farms are restricted*	4	8	0.17	0.02	1.10		
Restrictions on employee access to the site							
* Restricted after-hours only*	10	2	Ref.	-	-		
* Always restricted*	0	3	0.03	0.00	6.05		
* Not restricted*	1	5	0.04	0.00	0.41		
**Selection only by Random Forest**							
Sanitation procedure for employees and visitors entering the site							
* Unrestricted entry*	7	2	Ref.	-	-		
* One or more restrictions*	4	8	0.14	0.02	0.91		
Topography at the site							
* Flat*	2	7	Ref.	-	-		
* Hills or mountains*	9	3	10.49	1.57	105.00		
Disinfectant use on vehicles used to transport genetic animals							
* Hypochlorite (Clorox*,* Halazone*,* Chloramine-T) or peroxygen (Virkon) used*	0	2	Ref.	-	-		
* Iodine (Wescodyne*,* Premise*,* Iofec*,* Iosdyn*,* Losan)*,* or quaternary ammonium combinations (Synergize*,* Aseptol) used*	0	2	1.01	0.00	73.24		
* No disinfectant used or unknown*	2	3	3.63	0.12	111.68		
* Not Applicable (Select if vehicle used to transport animals is dedicated to this site)*	3	0	35.04	0.48	2435.04		
* Phenol-based compound (BioPhene*,* Environ*,* Tek-Trol*,* Laro*,* Lysol) or aldehydes (DC&R*,* Cidex*,* Formaldegen) used*	1	0	15.02	0.19	1236.03		
* Quaternary ammonium used (Roccal*,* Germex*,* Zephiran*,* Hi-Lethol*,* BioSentry)*	5	3	7.90	0.27	217.08		
**Selection only by LASSO**							
Boot and clothing restrictions between sites							
* No requirements*	7	4	Ref.	-	-		
* Required to change boots but not clothing*	3	1	1.71	0.15	41.39		
* Required to change clothing and boots*	1	5	0.11	0.01	1.04		
Feedback prior to entry							
* All sites naive*,* negative*,* or unknown/not positive*	6	9	Ref.	-	-		
* Positive stable*,* or active*	5	1	7.50	0.69	81.24		
Breeding females per on-site employee							
* 2.9–58.0*	7	4	Ref.	-	-		
* 58.1–167.0*	3	7	4.08	0.70	29.08		
**Association of herd status with other PRRSV measurements**							
PRRSV status, perception of the owner							
* Naive-entire herd never exposed*	1	7	Ref.	-	-		
* Negative but not naive (contains exposed animals)*	1	2	3.50	0.11	123.60		
* Positive stable*,* or active*	9	1	62.99	5.08	2525.01		
Time since the most recent PRRSV clinical outbreak							
* Never*	2	9	Ref.	-	-		
* Sometime ago*	9	1	40.49	4.34	1037.95		
N° of clinical outbreaks last 1 year							
*0*	6	9	Ref.	-	-		
*> 0*	5	1	7.50	0.91	164.02		

Ref.= Reference level; OR= odds ratio; 95% CI = 95% confidence interval; LL= lower limit; UL= upper limit. Firth’s correction was used for variables with quasi-complete separation

## Discussion

In this study, we identified three risk factors in Costa Rica that were significantly associated with the prevalence of PRRSV on farrow-to-finish pig farms: historical controlled exposure, sanitation before human entries, and topography of the area. Both historical controlled exposure and feedback practices before entry demonstrate that intentional pathogen introduction - even under controlled conditions - increases the risk of PRRSV positivity. These practices involve exposing herds or individual animals to the pathogen in a controlled manner, often using feedback materials such as infected tissues or fluids [26], with the aim to stimulate the animal’s immune systems and induce immunity without causing severe disease [27]. This approach carries inherent risks. The virus could spread beyond the intended group of animals, potentially leading to larger outbreaks, as reported in Korea and Japan for PEDv [28, 29]. Breaches in biosecurity measures or unforeseen environmental factors can exacerbate this risk. Furthermore, feedback practices may promote the co-circulation of multiple viral strains on a farm, particularly given PRRSV’s high mutation rate and genetic variability [30].

This was an observational study, and certain biosecurity-related factors, such as restrictions on employee movement between sites, were paradoxically associated with PRRSV seropositivity. This may reflect a response to prior outbreaks, where stricter biosecurity protocols were implemented after infection occurred. Nonetheless, limiting the movement of personnel between different sites remains critical to reduce the risk of inadvertently carrying the virus from one location to another [31]. In addition, our findings underscore the importance of implementing robust biosecurity measures, such as changing clothing and footwear and showering, and disinfectant use of vehicles, to prevent the transmission of pathogens between different production sites and other external sources [32]. Strategies to mitigate these risk factors include enforcing stricter biosecurity protocols, optimizing animal-to-staff ratios, and strengthening communication channels for dissemination of information on disease prevention and control [14].

Although hills and mountains can act as natural barriers and are often assumed to reduce the risk of PRRSV infection in pig herds, our study found that the probability of infection was higher for farms located in mountainous terrain. A previous study also associated increased risk of infection with the premontane forest ecozone [33]. This may be due to the more humid environment found in such areas, which could favor viral transmission or persistence. A key limitation of this study is the relatively small number of farms included in the modelling. Even though these were 21 of all 87 commercial farrow-to-finish farms in Costa Rica, this limits the generalizability of the results to the broader pig farming population.

To understand and predict PRRSV risk, we used two machine learning models with different algorithms: the Random Forest model and the LASSO model. Both models indicate possible associations, which can be used to generate hypotheses. The strength of the Random Forest model lies in its ability to handle complex interactions and non-linear relationships [18] and is well-suited for classification tasks. However, it may highlight variables that, while influential, are not necessarily emphasized in more parsimonious models. In contrast, the LASSO model focuses on identifying the most statistically significant predictors by applying regularization, thereby narrowing the analysis to key risk factors such as historically controlled exposure, employee visit restrictions, and site access restrictions. While LASSO offers a more simplified and interpretable set of predictors, it may overlook other potentially influential factors captured by the Random Forest model.

In summary, both models offer distinct advantages and limitations. The Random Forest model provides a comprehensive perspective by incorporating a broad range of variables, while the LASSO model narrows the focus to the most critical risk factors, enhancing interpretability. Integrating insights from both approaches can support the development of more targeted and effective strategies for PRRSV control.

## Supplementary Information

Below is the link to the electronic supplementary material.

Supplementary Material 1

Supplementary Material 2

Supplementary Material 3

## Data Availability

A data set was generated for this study. It is available as a complementary material.

## Abbreviations

PRRS: Porcine Reproductive Respiratory Syndrome
PRRSV: Porcine Reproductive Respiratory Syndrome Virus
LASSO: Least absolute shrinkage and selection operator
PADRAP: Production Animal Disease Risk Assessment Program

## References

[1] Keffaber KK. Reproductive failure of unknown etiology. Am Assoc Swine Pr Newsl. 1989;1:1–10.

[2] Lindhaus W. Lindhaus. Ratselhafte Schweinekrankheit. Prakt Tieraerz. 1991;72:423–5.

[3] Van Gucht S, Labarque G, Van Reeth K. The combination of PRRS virus and bacterial endotoxin as a model for multifactorial respiratory disease in pigs. Vet Immunol Immunopathol. 2004;102:165–78. 10.1016/j.vetimm.2004.09.006. 15507303 10.1016/j.vetimm.2004.09.006 PMC7112634

[4] Arruda AG, Friendship R, Carpenter J, Hand K, Ojkic D, Poljak Z. Investigation of the Occurrence of Porcine Reproductive and Respiratory Virus in Swine Herds Participating in an Area Regional Control and Elimination Project in Ontario, Canada. Transbound Emerg Dis. 2017;64:89–100. 10.1111/tbed.12343. 25766306 10.1111/tbed.12343

[5] Roepke D, Growing. Jul Losses from PRRS Cost Pork Producers $1.2 Billion Per Year, New Study Shows - Iowa State Research [Internet]. 2024 [cited 2025 Jul 7]. https://research.iastate.edu/2024/07/30/growing-losses-from-prrs-cost-pork-producers-1-2-billion-per-year-new-study-shows/. Accessed 7 2025.

[6] Meléndez-Arce R, Vargas-Leitón B, Steeneveld W, van Nes A, Stegeman JA, Romero- Zuñiga JJ. Stochastic model to assess bioeconomic impact of PRRS on pig farms in Costa Rica. Prev Vet Med. 2023;220:106032. 10.1016/j.prevetmed.2023.106032. 37778218 10.1016/j.prevetmed.2023.106032

[7] Pileri E, Mateu E. Review on the transmission porcine reproductive and respiratory syndrome virus between pigs and farms and impact on vaccination. Vet Res. 2016;47:108. 10.1186/s13567-016-0391-4. 27793195 10.1186/s13567-016-0391-4 PMC5086057

[8] Hancox L, Balasch M, Angulo J, Scott-Baird E, Mah CK. Comparison of viraemia and nasal shedding after PRRSV-1 challenge following vaccination with three commercially available PRRS modified live virus vaccines. Res Vet Sci. 2024;180:105416. 10.1016/j.rvsc.2024.105416. 39293105 10.1016/j.rvsc.2024.105416

[9] Bermúdez J. Estudio de Prevalencia de Anticuerpos a: Aujeszky, Peste Porcina Clásica, Gastroenteritis Transmisible, Coronavirus Respiratorio Porcino, Síndrome Reproductivo y Respiratorio Porcino en cerdos de Costa Rica. [Costa Rica]: Universidad Nacional; 1996.

[10] Nodelijk G, Nielen M, De Jong MCM, Verheijden JHM. A review of porcine reproductive and respiratory syndrome virus in Dutch breeding herds: population dynamics and clinical relevance. Prev Vet Med. 2003;60:37–52. 10.1016/S0167-5877(03)00081-3. 12900148 10.1016/s0167-5877(03)00081-3

[11] Meléndez R, Guzmán M, Jiménez C, Piche M, Jiménez E, León B, et al. Seroprevalence of porcine reproductive and respiratory syndrome virus on swine farms in a tropical country of the Middle Americas: the case of Costa Rica. Trop Anim Health Prod. 2021;53:441. 10.1007/s11250-021-02799-9. 34406521 10.1007/s11250-021-02799-9 PMC8373727

[12] Dewey C. PRRS in North America, Latin America, and Asia. Vet Res. 2000;31:84–5. 10.1051/vetres:2000015.

[13] Rodriguez-Vega V, Figueras-Gourgues S, Hernández-Caravaca I, Sala-Echave R, Diaz E. Use of PADRAP – Production Animal Disease Risk Assessment Program – in 91 farms in Spain.:1.

[14] Alarcón LV, Allepuz A, Mateu E. Biosecurity in pig farms: a review. Porc Health Manag. 2021;7:5. 10.1186/s40813-020-00181-z. 10.1186/s40813-020-00181-z PMC7780598 33397483

[15] Velasova M, Alarcon P, Williamson S, Wieland B. Risk factors for porcine reproductive and respiratory syndrome virus infection and resulting challenges for effective disease surveillance. BMC Vet Res. 2012;8:184. 10.1186/1746-6148-8-184. 23034160 10.1186/1746-6148-8-184 PMC3585917

[16] Holtkamp DJ, Lin H, Wang C, O’Connor AM. Identifying questions in the American Association of Swine Veterinarian’s PRRS risk assessment survey that are important for retrospectively classifying swine herds according to whether they reported clinical PRRS outbreaks in the previous 3 years. Prev Vet Med. 2012;106:42–52. 10.1016/j.prevetmed.2012.03.003. 22475927 10.1016/j.prevetmed.2012.03.003

[17] Silva GS, Corbellini LG, Linhares DLC, Baker KL, Holtkamp DJ. Development and validation of a scoring system to assess the relative vulnerability of swine breeding herds to the introduction of PRRS virus. Prev Vet Med. 2018;160:116–22. 10.1016/j.prevetmed.2018.10.004. 30388993 10.1016/j.prevetmed.2018.10.004

[18] Puhr R, Heinze G, Nold M, Lusa L, Geroldinger A. Firth’s logistic regression with rare events: accurate effect estimates and predictions? Stat Med. 2017;36:2302–17. 10.1002/sim.7273. 28295456 10.1002/sim.7273

[19] Mallioris P, Luiken REC, Tobias T, Vonk J, Wagenaar JA, Stegeman A, et al. Risk factors for antimicrobial use in Dutch pig farms: A cross-sectional study. Res Vet Sci. 2024;174:105307. 10.1016/j.rvsc.2024.105307. 38781817 10.1016/j.rvsc.2024.105307

[20] Breiman L. Random Forests. Mach Learn. 2001;45:5–32. 10.1023/A:1010933404324.

[21] Tibshirani R. Regression Shrinkage and Selection Via the Lasso. J R Stat Soc Ser B Stat Methodol. 1996;58:267–88. 10.1111/j.2517-6161.1996.tb02080.x.

[22] Friedman J, Hastie T, Tibshirani R. Regularization Paths for Generalized Linear Models via Coordinate Descent. J Stat Softw. 2010;33:1–22. 20808728 PMC2929880

[23] Meester M, Tobias TJ, van den Broek J, Meulenbroek CB, Bouwknegt M, van der Poel WHM, et al. Farm biosecurity measures to prevent hepatitis E virus infection in finishing pigs on endemically infected pig farms. One Health. 2023;16:100570. 10.1016/j.onehlt.2023.100570. 37363225 10.1016/j.onehlt.2023.100570 PMC10288132

[24] Lasso and Elastic-Net. Regularized Generalized Linear Models [Internet]. [cited 2024 Feb 15]. https://glmnet.stanford.edu/. Accessed 15 Feb 2024.

[25] R Core Team. (2024) R A Language and Environment for Statistical Computing. R Foundation for Statistical Computing. - References - Scientific Research Publishing [Internet]. [cited 2025 Oct 4]. https://www.scirp.org/reference/referencespapers?referenceid=3887298. Accessed 4 Oct 2025.

[26] Kumar D, Anderson Reever AV, Pittman JS, Springer NL, Mallen K, Roman-Sosa G, et al. Role of Pre-Farrow Natural Planned Exposure of Gilts in Shaping the Passive Antibody Response to Rotavirus A in Piglets. Vaccines. 2023;11:1866. 10.3390/vaccines11121866. 38140269 10.3390/vaccines11121866 PMC10748143

[27] Martínez-Lobo FJ, Díez-Fuertes F, Simarro I, Castro JM, Prieto C. The ability of porcine reproductive and respiratory syndrome virus isolates to induce broadly reactive neutralizing antibodies correlates with in vivo protection. front immunol [Internet]. Frontiers; 2021 [cited 2024 Aug 6];12. 10.3389/fimmu.2021.691145 10.3389/fimmu.2021.691145 PMC8350477 34381448

[28] Furutani A, Sekiguchi S, Sueyoshi M, Sasaki Y. Effect of intervention practices to control the porcine epidemic diarrhea (PED) outbreak during the first epidemic year (2013–2014) on time to absence of clinical signs and the number of dead piglets per sow in Japan. Prev Vet Med. 2019;169:104710. 10.1016/j.prevetmed.2019.104710. 31311633 10.1016/j.prevetmed.2019.104710

[29] Yamagami T, Miyama T, Toyomaki H, Sekiguchi S, Sasaki Y, Sueyoshi M, et al. Analysis of the effect of feedback feeding on the farm-level occurrence of porcine epidemic diarrhea in Kagoshima and Miyazaki Prefectures, Japan. J Vet Med Sci. 2021;83:1772–81. 10.1292/jvms.21-0343. 34615808 10.1292/jvms.21-0343 PMC8636866

[30] Linhares DCL, Cano JP, Torremorell M, Morrison RB. Comparison of time to PRRSv-stability and production losses between two exposure programs to control PRRSv in sow herds. Prev Vet Med. 2014;116:111–9. 10.1016/j.prevetmed.2014.05.010. 24931129 10.1016/j.prevetmed.2014.05.010

[31] Pitkin A, Deen J, Dee S. Further assessment of fomites and personnel as vehicles for the mechanical transport and transmission of porcine reproductive and respiratory syndrome virus. Can J Vet Res Rev Can Rech Veterinaire. 2009;73:298–302. PMC2757711 20046632

[32] Amass SF, Pacheco JM, Mason PW, Schneider JL, Alvarez RM, Clark LK, et al. Procedures for preventing the transmission of foot-and-mouth disease virus to pigs and sheep by personnel in contact with infected pigs. Vet Rec. 2003;153:137–40. 10.1136/vr.153.5.137. 12934795 10.1136/vr.153.5.137

[33] Alkhamis MA, Arruda AG, Vilalta C, Morrison RB, Perez AM. Surveillance of porcine reproductive and respiratory syndrome virus in the United States using risk mapping and species distribution modeling. Prev Vet Med. 2018;150:135–42. 10.1016/j.prevetmed.2017.11.011. 29169685 10.1016/j.prevetmed.2017.11.011

