# Supplementary material for: Uncovering biosecurity gaps: risk factors for PRRSV seropositivity in Costa Rican pig farms identified through machine learning
Source: Porcine Health Manag. 2026 Feb 21;12:16. doi: 10.1186/s40813-026-00495-4 (PMC13069804; doi:10.1186/s40813-026-00495-4)
Supplement: Supplementary file 1 — Supplementary Material 1 [file 40813_2026_495_MOESM1_ESM.docx]

**Supplementary Table 1. Variables to Assess Risk Factors for Porcine Reproductive and Respiratory Syndrome Virus (PRRSV) Introduction and Spreading into the Farm.**

| **Risk Factors** | **Explanation and Expected Answers** | **Risk Factors** | **Explanation and Expected Answers** |
| --- | --- | --- | --- |
| **Historical controlled exposure** | Refers to intentional exposure of pigs to PRRSV under controlled conditions to stimulate immunity. Answer: Whether or not the farm has practiced this. | **Production stages** | Describes the various production stages on the farm (e.g., farrowing, weaning). Answer: Outline the stages in operation. |
| **Feedback prior to entry** | Involves feeding tissues from infected pigs back to replacement pigs to stimulate immunity. Answer: Whether the farm uses this practice. | **Gestation housing** | The type of housing used for gestating sows (e.g., individual stalls, group housing). Answer: Specify housing conditions. |
| **Location of replacement animals** | Indicates where replacement animals are sourced from (e.g. within the farm, or another farm). Answer: Source of replacement animals. | **PRRSV status perception by the farmer** | The farm's current PRRSV status according to the owner (positive, negative, or unknown). Answer: Provide the PRRSV status of the farm. |
| **PRRSV status of replacements** | The PRRSV status of replacement animals before being brought into the herd, according to the farmer. Answer: Positive, negative, or unknown. | **Time since most recent PRRSV clinical outbreak** | How long it has been since the farm experienced a clinical outbreak of PRRSV. Answer: Time in weeks/months/years. |
| **Source of AI semen** | The origin of artificial insemination (AI) semen (whether from PRRSV-positive or negative sources). Answer: Source of semen and PRRSV status. | **Frequency of needle changes** | How often needles are changed when vaccinating or injecting animals. Answer: Frequency (e.g., after every animal, every few animals). |
| **Flow restrictions on vehicles** | Indicates whether vehicles are to follow a designated path within the farm to prevent cross-contamination. Answer: Yes or no. | **PRRSV status prior** | PRRSV status of animals before introducing them to the herd. Answer: Positive, negative, or unknown. |
| **Disinfectant use on vehicles used to transport animals** | Whether vehicles used to transport animals to market, or collection points are disinfected. Answer: Yes or no. | **Boot and clothing restrictions** | Indicates whether employees are required to change boots and clothing between sections of the farm. Answer: Yes or no. |
| **Disinfection of cab** | Refers to disinfecting the cabs of vehicles after transporting animals. Answer: Yes or no. | **Sanitation procedures for employees and visitors** | Procedures in place for cleaning and disinfecting employees and visitors entering the site. Answer: Description of the procedure. |
| **Route restrictions for genetic animal vehicles** | Indicates whether there are restrictions on the routes taken by vehicles transporting genetic animals. Answer: Yes or no. | **Employee restrictions on visits to other swine facilities** | Whether employees are restricted from visiting other swine facilities to prevent contamination. Answer: Yes or no. |
| **Use restrictions on vehicles for genetic animals** | Refers to whether there are specific use restrictions for vehicles that transport genetic animals. Answer: Yes or no. | **Number of sows** | The total number of sows on the farm. Answer: Number of sows. |
| **Disinfectant use on vehicles for genetic animals** | Indicates whether vehicles transporting genetic animals are disinfected. Answer: Yes or no. | **Average parity** | The average number of litters a sow has produced. Answer: Parity number. |
| **Type of load-out area** | Refers to the structure and design of the area where pigs are loaded for transport (e.g., enclosed, open). Answer: Type and description of the load-out area. | **Number of clinical outbreaks in the last year** | The number of PRRSV outbreaks the farm has experienced in the past year. Answer: Number of outbreaks. |
| **Flow of feed trucks** | Describes how feed trucks move around the farm and whether there are restrictions to limit cross-contamination. Answer: Yes or no. | **Source of breeding herds in last 2 years** | Indicates where the breeding herds were sourced from in the past two years. Answer: Provide the source of the herds. |
| **Flow of on-site employee vehicles** | Indicates whether there are any restrictions on how employee vehicles move on-site to avoid contamination. Answer: Yes or no. | **Breeding females per on-site employee** | The ratio of breeding females to employees on the farm. Answer: Number of breeding females per employee. |
| **Formal biosecurity training for new employees** | Whether new employees receive formal training on biosecurity measures. Answer: Yes or no. | **Pig density within 1 mile radius** | Describes the density of other pig farms/sites within 1 mile of the farm. Answer: High, medium, or low density. |
| **Procedures for introducing tools and supplies** | The biosecurity procedures in place when new tools and supplies are introduced onto the farm. Answer: Description of the procedure. |  |  |
| **Restrictions on employee access to the site** | Indicates whether there are restrictions on when employees can access different parts of the farm. Answer: Yes or no. |  |  |
| **Distance to nearest PRRSV-positive farm** | The distance from the farm to the nearest PRRSV-positive swine farm. Answer: Distance in miles or kilometers. |  |  |
| **Topography of the site** | Describes the physical landscape of the farm (e.g., flat, hilly). Answer: Describe the topography. |  |  |
| **Distance to major public road** | The distance to the nearest major road with frequent animal transportation. Answer: Distance in miles or kilometers. |  |  |
| **Distance to nearest swine market, slaughter plant, etc.** | The distance to the nearest swine market, slaughter plant, or collection point. Answer: Distance in miles or kilometers. |  |  |
