# Supplementary material for: Uncovering biosecurity gaps: risk factors for PRRSV seropositivity in Costa Rican pig farms identified through machine learning
Source: Porcine Health Manag. 2026 Feb 21;12:16. doi: 10.1186/s40813-026-00495-4 (PMC13069804; doi:10.1186/s40813-026-00495-4)
Supplement: Supplementary file 2 — Supplementary Material 2 [file 40813_2026_495_MOESM2_ESM.docx]

Info on Statistics

Random Forest

[Breiman L](https://en.wikipedia.org/wiki/Leo_Breiman) (2001). ["Random Forests"](https://doi.org/10.1023%2FA%3A1010933404324). [Machine Learning](https://en.wikipedia.org/wiki/Machine_Learning_(journal)).  [doi](https://en.wikipedia.org/wiki/Doi_(identifier)):[10.1023/A:1010933404324](https://doi.org/10.1023%2FA%3A1010933404324).

Random forest classification is an ensemble machine learning algorithm that uses multiple decision trees to classify data. The algorithm first takes a random sample of the farms and secondly a sample of a limited number of putative risk factors (train set). The samples were used in a tree-based method to find the most important predictive risk factors from the selected risk factors to classify the status of the farm. The most important predictive risk factors were then used to classify the farms not selected in the sample (out of the box) and compared to classify the true status of the farm. This cycle is rerun many times and the most important factors in each run were saved and finally summarized. The risk factors are than ordered based on the frequency they were selected in the run as most important factor.

LASSO

Tibshirani, Robert (1996). "Regression Shrinkage and Selection via the lasso". Journal of the Royal Statistical Society. Series B (methodological). 58 (1). [doi](https://en.wikipedia.org/wiki/Doi_(identifier)):[10.1111/j.2517-6161.1996.tb02080.x](https://doi.org/10.1111%2Fj.2517-6161.1996.tb02080.x)

LASSO regression use a regularization technique and performs [variable selection](https://en.wikipedia.org/wiki/Variable_selection) in order to enhance the prediction accuracy and interpretability of the resulting [statistical model](https://en.wikipedia.org/wiki/Statistical_model) which prevents overfitting due to a large number of variables in the model. The lasso method assumes that the coefficients of the linear model are sparse, meaning that few of them are non-zero. The algorithm shrinks stepwise the smaller estimates for variables to 0 optimizing the remaining coefficients based on the deviance improving prediction accuracy. Finally the subset of variables is chosen with the smallest deviance.
